# A superior drug carrier – aponeocarzinostatin in partially unfolded state fully protects the labile antitumor enediyne

**DOI:** 10.1186/1423-0127-16-48

**Published:** 2009-05-23

**Authors:** Aranganathan Shanmuganathan, Thallapuranam Krishnaswamy Suresh Kumar, Chiy-Mey Huang, Chin Yu, Der-Hang Chin

**Affiliations:** 1 Department of Chemistry, National Chung Hsing University, Taichung, Taiwan 40227, Republic of China; 2 Department of Chemistry, National Tsing Hua University, Hsinchu, Taiwan 30013, Republic of China; 3 Department of Chemistry and Biochemistry, University of Arkansas, Fayetteville, Arkansas 72701, USA

## Abstract

**Background:**

Neocarzinostatin is a potent antitumor drug consisting of an enediyne chromophore and a protein carrier.

**Methods:**

We characterized an intermediate in the equilibrium unfolding pathway of aponeocarzinostatin, using a variety of biophysical techniques including 1-anilino-8-napthalene sulfonate binding studies, size-exclusion fast protein liquid chromatography, intrinsic tryptophan fluorescence, circular dichroism, and ^1^H-^15^N heteronuclear single quantum coherence spectroscopy.

**Results:**

The partially unfolded protein is in molten globule-like state, in which ~60% and ~20% tertiary and secondary structure is disrupted respectively. Despite lacking a fully coordinated tertiary structure for assembling a functional binding cleft, the protein in molten globule-like state is still able to fully protect the labile chromophore. Titration of chromophore leads the partially denatured apoprotein to fold into its native state.

**Conclusion:**

These findings bring insight into conserving mechanism of neocarzinostatin under harsh environment, where even the partially denatured apoprotein exhibits protective effect, confirming the superiority of the drug carrier.

## Background

Neocarzinostatin (NCS) is the most studied member within the family of natural enediyne-based chromoproteins with potent anti-tumor activity [1,2]. Holoneocarzinostatin (holoNCS) drug consists of a biologically active chromophore (NCS-Chr) that is non-covalently bound to a carrier apoprotein (apoNCS). NCS-Chr is very labile and can be inactivated quickly when it is not associated with apoNCS [1,3,4]. To carry out the protection role, a regular drug carrier protein must fold properly to form a well-defined specific binding cleft before it can accommodate the ligand molecule. Here we report an interesting observation that apoNCS in its partially unfolded intermediate state is able to efficiently bind and protect the labile NCS-Chr. Elucidation of the protein folding with respect to chromophore protection could serve as a starting point for rational drug carrier design strategies.

**Figure 1:**
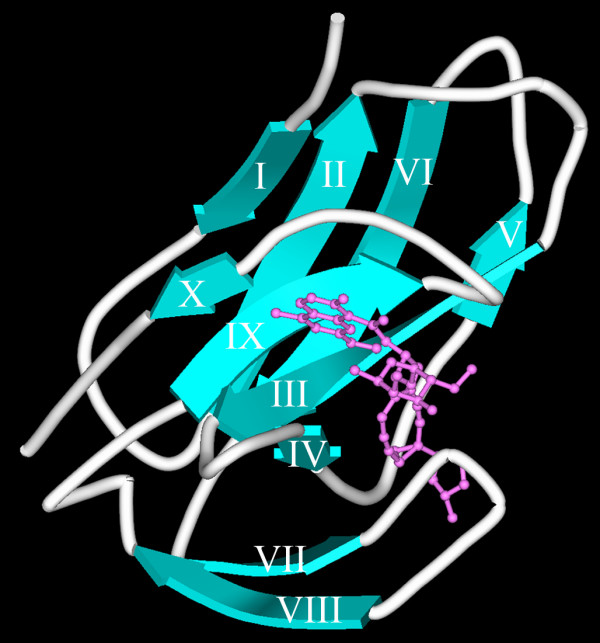
**Three-dimensional model of holoNCS in an aqueous environment at pH 7**. The model was modified [5] from Brookhaven pdb.1nco.ent file. Chromophore is shown as a ball-and-stick representation (magenta). The all β-sheet holoNCS (cyan) consists of an antiparallel β-barrel and a small double-stranded β-sheet domain.

ApoNCS is an all β-sheet protein (~11 kDa) consisting of an antiparallel β-barrel and a β-sheet domain (Fig. 1, NCS model in an aqueous environment at pH 7 [5]). Folding and unfolding pathways of this small all β-sheet protein have been an interesting topic recently studied by various methods [6-11]. Results from some studies show that the folding/unfolding of apoNCS may not follow a simple two-state model, instead, stable folding intermediates may be involved in the transition pathways [9-11]. Nonetheless, detailed characterization of stable apoNCS folding/unfolding intermediates has not been reported.

Structural characterization of intermediates that populate in the folding/unfolding process is crucial to understand the protein folding mechanism. Equilibrium and kinetic intermediates have been identified in the unfolding/refolding reactions of several proteins [12-15]. The best studied intermediate is the molten globule (MG) state [16,17]. The MG states are believed to be general folding intermediates because they populate both in the equilibrium and kinetic folding/unfolding pathways [16,17]. In the present study, we identified and characterized a stable intermediate in the guanidine hydrochloride (GdnHCl)-induced equilibrium unfolding pathway of apoNCS. Intermediate accumulates maximally in 1.2 M GdnHCl under acidic condition and has structural properties resembling that of a MG-like state. To gain insights into the biological role of apoNCS as a drug carrier, the interaction of MG-like intermediate with NCS-Chr was investigated.

## Methods

### Materials

NCS powder consisting apoNCS and NCS-Chr in a 1:1 molar ratio was a gift from Kayaku Co., Ltd., Itabashi-Ku, Tokyo, Japan. Extracted NCS-Chr was obtained after repeated methanol extractions of lyophilized NCS stock (0.5 mM, as determined by ε_340 _= 10,800 M^-1 ^cm^-1^) in 20 mM sodium citrate (pH 4), following the previously described method [18]. Extracted NCS-Chr was stored at -80°C in amber glass vials. Integrity and concentration of NCS-Chr were examined by UV spectroscopy and HPLC analysis. Labeled ^15^NH_4_Cl and D_2_O were purchased from Cambridge Isotope Laboratories (Andover, MA, USA). All other chemicals used were of high quality analytical grade. All experiments were performed in 10 mM phosphate buffer (pH 3) at 25°C.

### Expression and purification of apoNCS

Recombinant apoNCS was overexpressed and purified by the procedure as described [19]. Homogeneity of the protein was examined by UV, HPLC and SDS-PAGE. Protein yield was about 2 mg/L. Molecular mass of the purified protein was verified using ESI-Mass analysis.

### Preparation of isotope-enriched apoNCS

Uniform ^15^N labeling was achieved by the established procedure [19]. *E. coli *BL21 Codon Plus strain (Stratagene, La Jolla, CA, USA) carrying the apoNCS gene was cultured in M9 minimal medium containing ^15^NH_4_Cl supplemented with vitamin B_1_. Final yield of the ^15^N-labeled protein was about half of the corresponding unlabeled protein expressed in LB medium.

### Steady-State fluorescence measurements

Fluorescence spectra were collected using a Hitachi F-4500 spectrofluorimeter at a resolution of 2.5 or 10 nm. For GdnHCl-induced unfolding study, excitation wavelength was set at 280 nm. Intrinsic fluorescence measurements were made at 25°C. Binding affinity of 1-anilino-8-napthalene sulfonate (ANS) to apoNCS at various concentrations of GdnHCl was monitored in a wavelength range of 375 to 625 nm using an excitation wavelength at 355 nm. Excitation and emission bandwidths were set at 5 nm. Concentration of ANS and protein was 100 μM and 10 μM respectively. All samples were prepared in 10 mM phosphate buffer at pH 3.

### Circular dichroism (CD) spectroscopy

All CD measurements are carried out on a Jasco J-715 spectropolarimeter (Tokyo, Japan) equipped with a circulating water bath (Neslab, model RTE-140) (Portsmouth, NH, USA). Measurements were made using a 0.1 cm path-length water-jacketed quartz cell. Each spectrum represents an average of 30 scans with a scan speed of 50 nm/min. Concentration of the protein used was 15 μM. Background corrections were made in all spectra. Bandwidth was set to 1 nm and all spectra were acquired at 25°C.

### Size-exclusion chromatography (SEC)

Gel-filtration experiments were carried out at 25°C on a superdex-100 column using a Pharmacia AKTA FPLC chromatographic device (GE Healthcare Bio-Sciences AB, Uppsala, Sweden). Column was equilibrated with 2 bed volumes of 10 mM phosphate buffer (pH 3) containing appropriate concentrations of GdnHCl at a flow rate of 1 ml/min. Concentration of protein used for each analysis was approximately 250 μg/ml (dissolved in appropriate concentrations of GdnHCl). Protein elution was monitored by UV absorbance at 280 nm.

### Thermal denaturation experiments

Thermal stability of apoNCS in 10 mM phosphate buffer (pH 3) in the presence or absence of 1.2 M GdnHCl was monitored by far-UV CD at 224 nm. Changes in ellipticity with temperature were followed from 5–91°C at an increment of 3°C. Experiments were performed using a water-jacketed cell connected to a thermal circulator equipped with a microprocessor and temperature sensor. Protein sample (25 μM) was allowed to equilibrate for 10 minutes at each temperature before data acquisition.

### NMR experiments

The NMR experiments were carried out on a Bruker DMX 600 MHz NMR spectrometer (Rheinstetten, Germany) at 25°C. A 5 mm inverse probe with a self-shielded z-gradient was used to obtain all gradient-enhanced ^1^H-^15^N HSQC spectra [20,21]. ^15^N decoupling during acquisitions was achieved using the GARP sequence [22]. Total of 2048 complex data points were collected in the ^1^H-dimension of the ^1^H-^15^N HSQC experiments. In the indirect ^15^N-dimension spectra, 512 complex data points were collected. The HSQC spectra were recorded by 32 scans at all concentrations of GdnHCl. ^15^N chemical shifts were referenced using consensus ratio of 0.0101329118. All spectra were processed on a Silicon Graphics workstation using XWINNMR and Sparky softwares.

### Binding experiments

Binding experiments were performed in 10 mM phosphate buffer (pH 3) by incubating 20 μM apoNCS (in the presence or absence of 1.2 M GdnHCl) with NCS-Chr at 1:1 molar ratio for 30 minutes at 25°C. The final methanol content introduced from NCS-Chr stock was kept minimal to about 4% (v/v). Analyses of the protein-bound NCS-Chr after binding experiments were performed through a Waters μ-Bondapak reverse phase C_18 _column by a Waters Millennium HPLC equipped with a model 600E solvent delivery system, a 996 photodiode array detector and either a Waters 474 or a Jasco FP-1520 fluorescence detector following previously described method [23].

## Results

### GdnHCl-induced unfolding of apoNCS does not follow a two-state model

**Figure 2:**
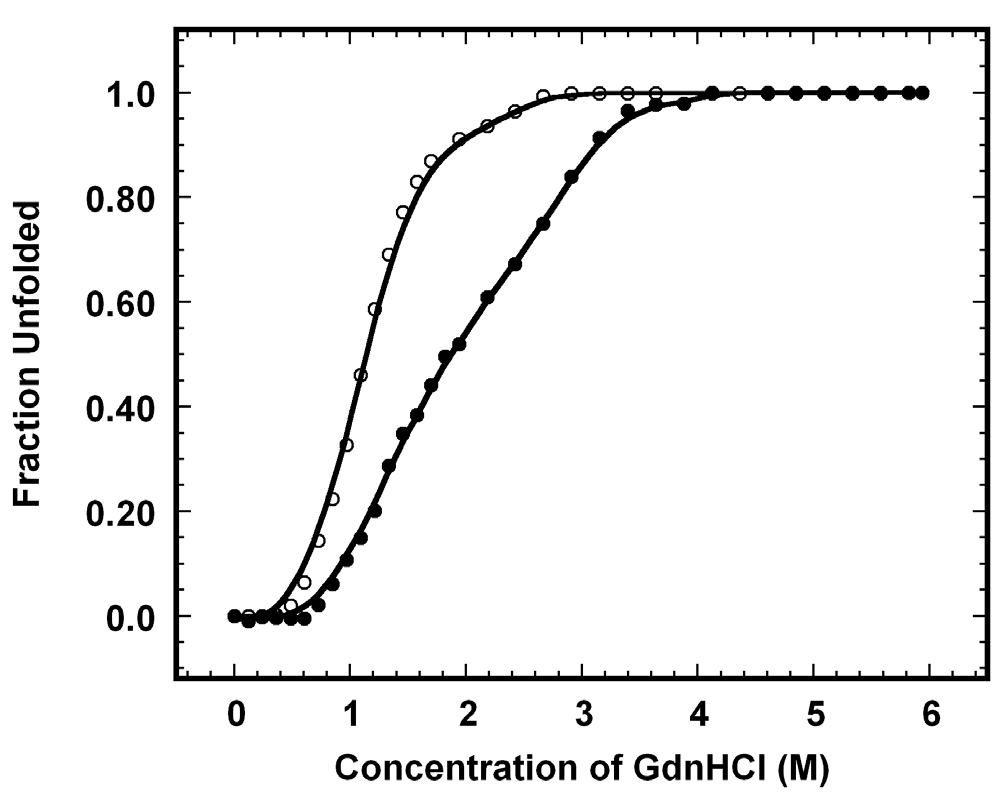
**Fraction of unfolded apoNCS at various concentrations of GdnHCl**. The GdnHCl-induced unfolding at 25°C was monitored by changes in fluorescence emission at 340 nm (excited at 280 nm) (open circle) and ellipiticity at 224 nm (black circle). The concentration of apoNCS used in the fluorescence and far-UV CD experiments was 5 and 15 μM, respectively. Samples were prepared in 10 mM phosphate buffer at pH 3.

GdnHCl-induced equilibrium unfolding of apoNCS at pH 3 was monitored by changes in intrinsic tryptophan fluorescence and far-UV CD at 224 nm. Circular dichroism spectrum of the all β-sheet apoNCS protein is somewhat atypical [24] with a large positive maximum at 224 nm and small negative minimum centered at 212 nm [7]. Fluorescence and far-UV CD spectral probes have been shown to reliably report the gross tertiary and secondary structural changes that possibly occur during the unfolding of apoNCS [11]. Fig. 2 shows that GdnHCl-induced equilibrium unfolding of apoNCS under acidic condition (pH 3) monitored by changes in intrinsic tryptophan fluorescence is completely reversible. The fluorescence unfolding profile of apoNCS shows that the protein starts to unfold in 0.6 M GdnHCl. Unfolding of the protein is completed beyond 3 M GdnHCl (Fig. 2). C_m _(concentration of GdnHCl at which 50% of the protein molecules are in denatured state) and '*m*' (measure of cooperativity of unfolding process) are 1.1 ± 0.1 M and 7.3 ± 0.7 kJ mol^-1 ^M^-1 ^respectively. Change in free energy of unfolding obtained in the absence of the denaturant [ΔG (H_2_O)] is estimated to be 8.5 ± 0.1 kJ mol^-1^. Interestingly, the GdnHCl-induced unfolding curve obtained by measuring ellipticity changes at 224 nm is not superimposable with that obtained by monitoring the changes in intrinsic tryptophan fluorescence. These results possibly suggest that the unfolding of apoNCS may not follow a two-state model, and may involve accumulation of stable intermediate states (Fig. 2). C_m_, '*m*' and [ΔG (H_2_O)] estimated from the unfolding profile obtained using far-UV CD are 1.8 ± 0.1 M, 4.4 ± 0.2 kJ mol^-1 ^M^-1^and 8.6 ± 0.5 kJ mol^-1 ^respectively.

### A stable equilibrium unfolding intermediate of apoNCS

**Figure 3:**
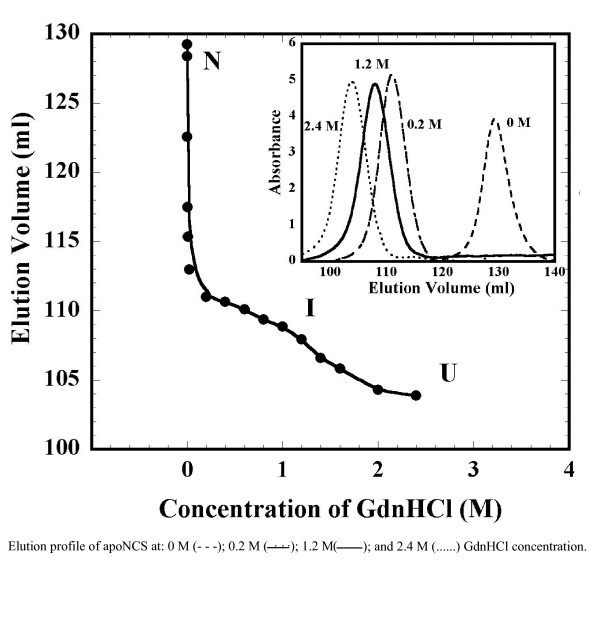
**GdnHCl-induced unfolding of apoNCS demonstrated by SEC analyses**. The changes in elution volume of apoNCS at pH 3 were measured at various concentration of GdnHCl. Transition from native (N) to intermediate (I) state occurs in the first phase (0–1.2 M GdnHCl). Transition from intermediate (I) to denatured (D) state occurs in the second phase beyond 1.3 M GdnHCl.

Size-exclusion chromatography (SEC) is a useful technique to acquire information on integral changes of molecular dimensions under the effect of the denaturant [25,26]. This technique has been successfully used to identify and obtain hydrodynamic data on stable intermediates in the folding/unfolding pathway of proteins [25]. ApoNCS in 10 mM phosphate buffer (pH 3) in the absence of GdnHCl elutes as a single peak with an elution volume of ~129 ml (Fig. 3). On increasing the concentration of GdnHCl from 0 to 0.2 M, the elution volume of the protein peak sharply decreases to about ~111 mL. Interestingly, elution volume of the protein does not significantly change when the GdnHCl concentration is increased from 0.4 M to 1.2 M (Fig. 3). A clear plateau can be observed in the plot of elution volume versus concentration of GdnHCl suggesting the accumulation of a stable equilibrium intermediate in this range of denaturant concentration. Beyond 1.2 M GdnHCl, elution volume of the protein changes drastically and profile eventually reaches a plateau at 2 M GdnHCl. It should be mentioned that apoNCS elutes as a single peak at various concentrations of GdnHCl indicating that the protein exists as a single population at all concentrations of the denaturant (Fig. 3, inset). Uversky [26], using size-exclusion chromatography to study the denaturant-induced equilibrium unfolding profile of proteins, showed that proteins such as β-lactamase, bovine carbonic anhydrase and β-lactoglobulin, which denature through a MG-like intermediate, also elute as single peaks at all concentrations of the denaturant [26]. The results of the SEC-FPLC experiment clearly show that the GdnHCl-induced unfolding of apoNCS proceeds through the accumulation of a stable intermediate at around 1.2 M GdnHCl at pH 3 (Fig. 3). At pH above 3 no stable intermediate was observed. The first phase of apoNCS unfolding between 0–1.2 M GdnHCl appears to represent an equilibrium transition between the native and the intermediate state.

### Partially unfolded apoNCS intermediate resembles MG-like state

**Figure 4:**
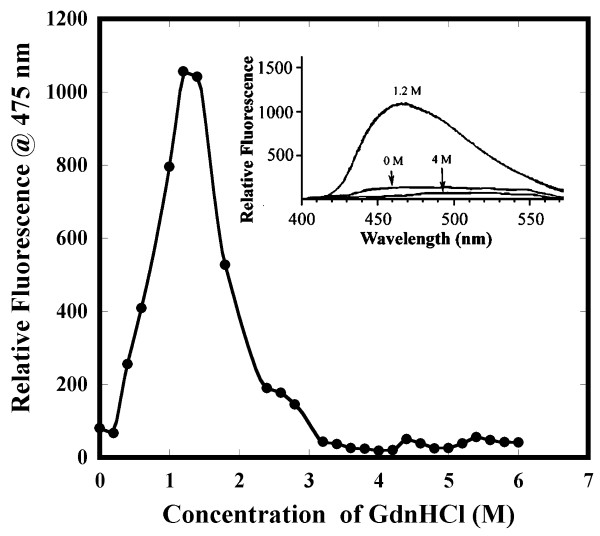
**Binding of ANS to apoNCS at various concentrations of GdnHCl**. Fluorescent emission intensity of ANS (100 μM) in the presence of apoNCS (10 μM) was followed at 25°C in different concentration of GdnHCl using an excitation wavelength at 355 nm. Samples were prepared in 10 mM phosphate buffer at pH 3. Inset shows emission spectra of ANS with apoNCS in native (N, in 0 M GdnHCl), intermediate (I, in 1.2 M GdnHCl) and denatured (D, in 4 M GdnHCl) state.

Under optimal conditions (pH 3, 1.2 M GdnHCl) where the stable unfolding intermediate of apoNCS accumulates, considerable percentage (about 80%) of the secondary structure remains undisrupted, whereas 60% of the tertiary structure is disrupted (Fig. 2). This is a good indication that the stable intermediate could be in MG-like state. ANS is a fluorescent dye that binds to the hydrophobic regions of proteins [27] and this fluorescent probe has been immensely useful in the identification of equilibrium intermediates such as MG states. MG intermediates usually display significant exposure of hydrophobic cores to the solvent. Therefore, ANS binds strongly to MG states and fluoresces intensely [28,29]. The dye generally exhibits weak binding affinity to the native and denatured proteins [28]. The binding affinity of apoNCS to ANS at various concentrations of GdnHCl was monitored by changes in the emission intensity at 475 nm (Fig. 4). The ANS emission intensity upon binding to apoNCS in 1.2 M GdnHCl is significantly higher than that observed with the protein in its folded state (Fig. 4). Further increase in the concentration of GdnHCl (beyond 1.2 M) results not only in the progressive decrease in the emission intensity but also in a continuous red shift in the emission maxima. The protein in its unfolded conformations, at and beyond 3 M GdnHCl, exhibits weak binding to ANS (Fig. 4). Thus, the results of ANS binding and SEC-FPLC experiments, analyzed in conjunction, clearly suggest that a MG-like intermediate accumulates maximally in 1.2 M GdnHCl.

### Thermal stability of MG-like intermediate of apoNCS

**Figure 5:**
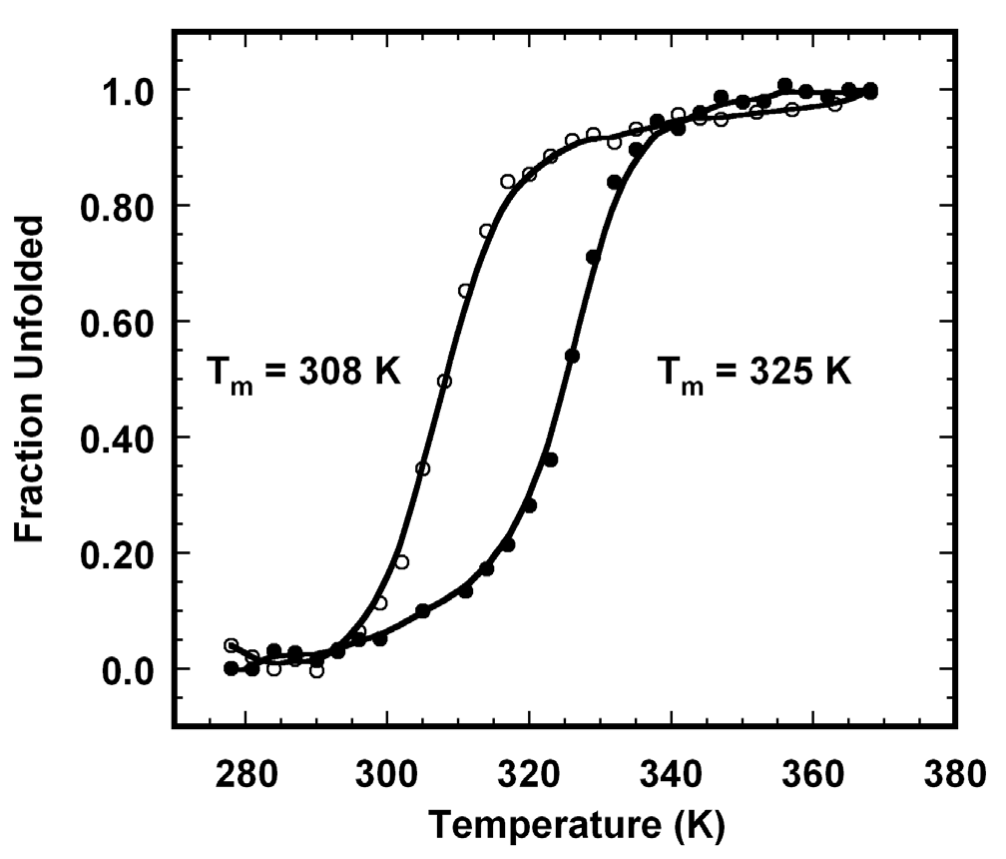
**Thermal unfolding of apoNCS at the intermediate state**. Heat-induced unfolding of apoNCS was monitored by changes in ellipticity at 224 nm in the absence (black circle) and presence (open circle) of 1.2 M GdnHCl. ApoNCS samples (25 μM) were prepared in 10 mM phosphate buffer at pH 3.

It is important to understand the stability of the equilibrium unfolding intermediate states in relation to the folded state of the protein. In this context, we carried out thermal denaturation of apoNCS in the presence and absence of 1.2 M GdnHCl at pH 3 (Fig. 5). Thermal unfolding monitored by ellipticity changes at 224 nm shows that apoNCS at pH 3 unfolds to denatured state at temperatures beyond 62°C (Fig. 5). Apparent T_m _(temperature at which 50% of the protein molecules exist in the denatured state) of the unfolding reaction is about 52 ± 1°C. In marked contrast, the MG-like state of the protein at the same pH level in 1.2 M GdnHCl unfolds almost completely beyond 50°C (Fig. 5). T_m _of the MG-like state to denatured state of apoNCS is 35 ± 1°C. These results suggest that the protein in the MG-like state is significantly less stable than the folded state. It appears that some of the interactions stabilizing the compactly folded conformation are disrupted in MG-like state.

### NMR studies on structural changes in MG-like state of apoNCS

**Figure 6:**
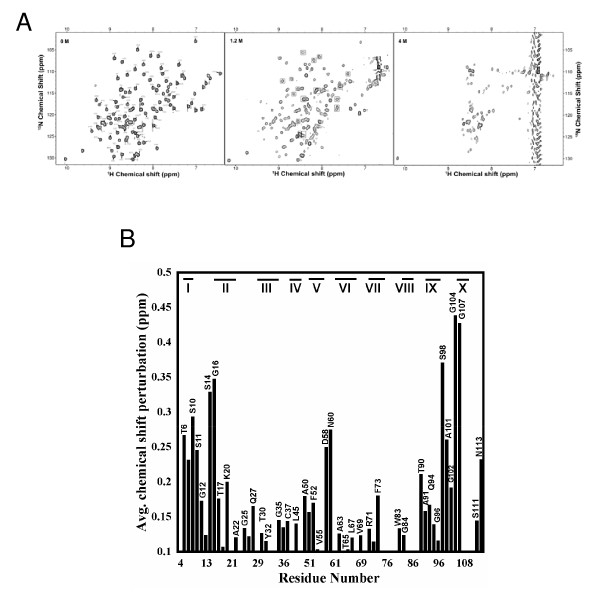
**NMR analysis of apoNCS in various concentrations of GdnHCl**. (A) ^1^H-^15^N HSQC spectra of apoNCS obtained at 25°C in 0, 1.2, and 4 M GdnHCl. Residues which show maximum perturbation in the intermediate state in 1.2 M GdnHCl are boxed. (B) The weighted average (of ^15^N and ^1^H) chemical shift perturbation of residues in 1.2 M GdnHCl. ApoNCS samples (720 μM) were prepared in 10 mM phosphate buffer (pH 3) in 90% H_2_O and 10% D_2_O. The β-strands in the protein are shown in roman numerals (see Fig. 1).

Two-dimensional ^1^H-^15^N heteronuclear single quantum coherence (HSQC) NMR spectroscopy serves as a fingerprint of the conformational state of a protein. It provides residue level information on the structural changes that possibly occur during unfolding/folding processes. We synthesized and purified ^15^N-labeled apoNCS and monitored GdnHCl-induced unfolding by ^1^H-^15^N chemical shift perturbation. Heteronuclear correlation experiments have been shown to be very sensitive because of high magnetization transfer between directly bonded nuclei. Fig. 6A shows that 2D ^1^H-^15^N HSQC spectrum of apoNCS at pH 3 in the absence of GdnHCl is well-dispersed, which is a characteristic feature of a folded protein. Striking differences can be discerned in the HSQC spectra obtained in the absence and presence of 1.2 M GdnHCl. Many cross peaks in HSQC spectrum of the protein in 1.2 M GdnHCl are broadened, suggesting the overall flexibility of residues in the intermediate state (in 1.2 M GdnHCl) is significantly higher than that in the folded state (Fig. 6A). Though it is not easy to identify residues in the partially denatured state, we attempted to make an assignment by recording a series of HSQC NMR spectrum of apoNCS at pH 5 to 3 with 0.5 decrement of pH level. ^1^H and ^15^N backbone chemical shifts of native apoNCS at pH 5 were assigned based on the reported values (105 of 113 residues of apoNCS were reported) [30]. Once the cross peaks of apoNCS spectrum obtained at pH 3 were assigned, they were compared with those recorded at pH 3 in the presence of GdnHCl. By stepwise comparison, we identified 70 residues in the spectrum of MG-like state of apoNCS. The weighted average (of ^15^N and ^1^H) chemical shift perturbation ((Δδ) = [(δH)^2 ^+ 0.2 (δ^15^N)^2^)]^1/2^) of residues in MG-like state of apoNCS is shown in Fig. 6B, in which the unassigned residues (residues 1–3, 5, 8, 9, 15, 19, 24, 28, 31, 38–44, 46, 48, 49, 53, 54, 56, 59, 62, 66, 75, 78, 79, 81, 82, 87, 89, 92, 93, 97, 99, 100, 103, 105, 106, 112) as well as the residues with perturbation below 0.1 ppm (the threshold of the average minimal perturbation) were eliminated. The result clearly shows that residues at N- and C-termini of the protein are significantly perturbed in the MG-like state (Fig. 6B). Most of the residues that show significant chemical shift changes or peak broadening appear to be those not involved in the secondary structure formation (Fig. 6A and 6B). HSQC spectra of apoNCS acquired beyond 4 M GdnHCl show significant decrease in chemical shift dispersion in ^1^H dimension indicating complete unfolding of the protein (Fig. 6A). The results of the NMR experiments suggest that although the interactions at loop regions and both N- and the C-termini of the protein are perturbed, the secondary structural elements in the protein appear to be mostly unaffected in the equilibrium intermediate in 1.2 M GdnHCl.

### ApoNCS in partially unfolded MG-like state fully protects labile NCS-Chr

NCS manifests its antineoplastic effects through damage to the cellular genome [1]. The very potent cytotoxic activities come from the enediyne warhead NCS-Chr, which is very labile by itself [3]. At pH 8, the life time of free NCS-Chr in an aqueous environment is only few seconds [4]. Binding with apoNCS increases the life time of NCS-Chr to several thousand folds [4]. Thus, the degree of protection against spontaneous NCS-Chr degradation can serve as a good probe to assess the binding affinity towards NCS-Chr [4,31]. To examine whether apoNCS in MG-like state binds to NCS-Chr, an aliquot of NCS-Chr was incubated with 1:1 molar ratio of apoNCS at pH 3 and 25°C for 30 min in the presence and absence of 1.2 M GdnHCl. The remaining amount of intact NCS-Chr was analyzed by HPLC following the previously established method [23]. The results clearly show that NCS-Chr was fully protected in the presence of either folded or MG-like state of apoNCS (Table 1). Evidently, the MG-like state of apoNCS in 1.2 M GdnHCl retains its full ability to bind to its enediyne chromophore.

**Table 1:** Protection of NCS-Chr against degradation by binding with MG-like state of apoNCS

Sample contents	Remaining % of NCS-Chr^*a*^
NCS-Chr	0%
NCS-Chr + 1.2 M GdnHCl	13 ± 4%^*b*^
holoNCS (NCS-Chr + 1:1 folded state of apoNCS)	100 ± 4%
NCS-Chr + 1:1 MG-like state of apoNCS (in 1.2 M GdnHCl)	101 ± 4%^*c*^

^*a *^Samples containing 20 μM NCS-Chr were incubated with folded or MG-like state of apoNCS in 10 mM phosphate aqueous buffer at pH 3 and 25°C for 30 min. Remaining amount of intact NCS-Chr was analyzed by HPLC.

^*b *^Most NCS-Chr was degraded in acidic aqueous condition in the presence of 1.2 M GdnHCl. The small remaining amount of the NCS-Chr exhibited additional forms. About 35 ± 10% of the remaining NCS-Chr was converted into chlorohydrin form of the chromophore. It is known that the chlorohydrin derivative of NCS-Chr can be obtained by treatment of HCl [2]. Presumably, excess amount of GdnHCl triggers the conversion process.

^*c *^No chlorohydrin conversion could be found in the remaining NCS-Chr in the presence of MG-like state of apoNCS. Presumably, binding of apoNCS prevents the chromophore conversion reaction triggered by excess amount of GdnHCl.

### MG-like state of apoNCS resumes its folded state after binding to NCS-Chr

**Figure 7:**
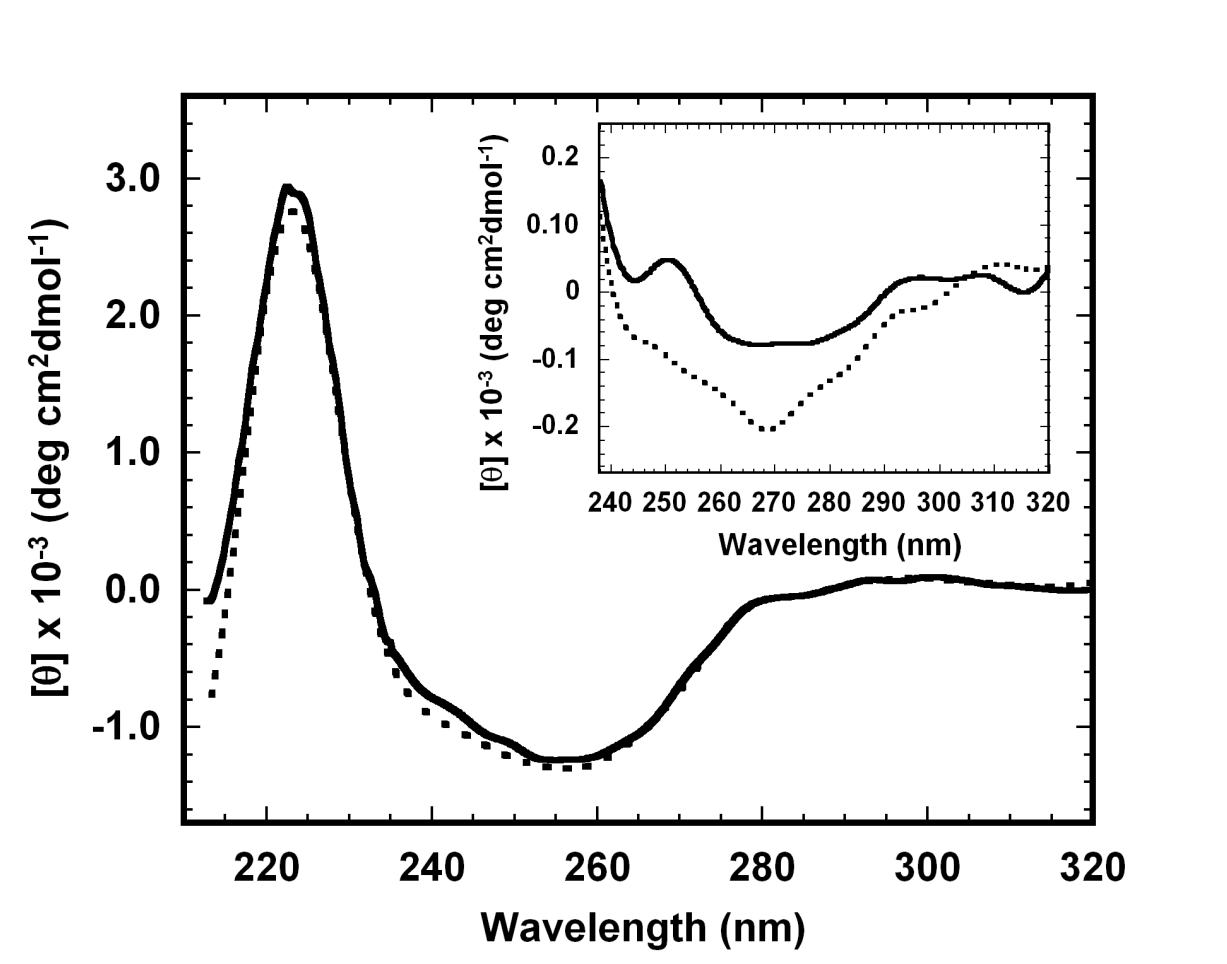
**CD spectra of apo- and holoNCS in MG-like and native state**. CD spectra of the natural holoNCS (dotted line) in 10 mM phosphate buffer (pH 3) and the constituted holoNCS of NCS-Chr and MG-like state of apoNCS (full line) in 1.2 M GdnHCl (pH 3). MG-like state of apoNCS for constitution was prepared by incubating 15 μM apoNCS with 1.2 M GdnHCl in 10 mM phosphate (pH 3) at 25°C for 30 min. Constitution was performed prior to CD measurement by adding 1:1 molar ratio of methanol extracted NCS-Chr into aqueous solution containing MG-like state of apoNCS. Final methanol content was kept minimal to about 4% (v/v).*Inset*: Near-UV CD spectrum of apoNCS in MG-like state (full line) (in the presence of 1.2 M GdnHCl (pH 3)) and in folded state (dotted line) (in the absence of GdnHCl in 10 mM phosphate buffer (pH 3)). All CD spectra were recorded in mean residue ellipticity at 25°C.

Titration of NCS-Chr into native state apoNCS can produce drastic changes in the near-UV CD spectrum [19,32]. Binding of 1:1 molar ratio of NCS-Chr with native apoNCS produces a prominent negative ellipticity peak minimum at 255 nm, which is a characteristic feature of the native form of holoNCS. Interestingly, titration of 1:1 molar ratio of NCS-Chr into MG-like state of apoNCS in 1.2 M GdnHCl at pH 3 produces identical CD spectrum to that of the native holoNCS (Fig. 7). The results unambiguously suggest that NCS-Chr not only can bind to apoNCS in partially unfolded MG-like state but also help the protein fold back into its native state. Without NCS-Chr, the near-UV CD spectroscopy of the MG-like state of apoNCS shows that 60% of the tertiary structure is disrupted (Fig. 7, inset). The observation is consistent with that from intrinsic tryptophan fluorescence study (Fig. 2).

## Discussion

Understanding the mechanism by which a protein folds from denatured state into its unique native three-dimensional structure is an important problem in molecular biology [33,34]. ApoNCS, being a model for small all β-sheet proteins, has been extensively investigated for its folding/unfolding pathways [6-11]. Formation of intermediate state has been suggested when apoNCS is treated with 45% trifluoroethanol at pH 5 [11]. Under aqueous condition, we demonstrated that the thermal unfolding of apoNCS at pH 7 follows a two-state mechanism [7]. On the contrary, small-angle neutron scattering studies on apoNCS at pH 7 reveal that there could be several discrete intermediate species at equilibrium populated in the unfolding pathways [9,10]. The distribution of those substates shows various degrees of residual structures and appears to be temperature or solvent dependent. The major species present during the transition could be considerably unstructured, which may make the thermal unfolding transition look like a two-state transition [9]. This accounts for the failure of identifying a well-defined intermediate state in the unfolding pathway of apoNCS at pH 7 [10]. In the present study, we identified and characterized a stable MG-like state in the GdnHCl-induced unfolding pathway of apoNCS only at a rather acidic pH (pH 3). At pH 5, where apoNCS is stable at room temperature [7], we have shown that apoNCS is highly resistant against denaturants [35]. The transitions of apoNCS induced by GdnHCl can not be complete even at the highest concentration of the denaturant. Here we also screened GdnHCl-induced unfolding pathways of aqueous apoNCS at 25°C over a pH range of 3 to 9 (data not shown). We could not observe any stable intermediates except at pH 3. Conceivably, the chances of characterizing a stable intermediate are limited by conditions. We wish the present characterization of a MG-like state intermediate would provide some inputs for further understanding of the complex folding/unfolding mechanism of apoNCS.

Potent anti-neoplastic activity of NCS comes from its NCS-Chr, and apoNCS serves its functional role as a carrier and protector [1,3,4]. Without apoNCS, NCS-Chr is very labile and can be inactivated quickly by bases, light, heat, and chemicals such as cellular thiols [1,3,4]. When NCS-Chr and apoNCS are biosynthesized from specific gene clusters that produce NCS, the peptide chain of apoNCS needs to fold properly and efficiently to form specific binding cleft for accommodation of the chromophore. Statistical studies have suggested that proteins with more complex topologies such as β-sheets usually fold more slowly than proteins with α-helices [36]. ApoNCS being an all β-sheet protein, its topology is not favorable for fast folding. Although the role of ligand in protein folding is not well understood, there are studies showing that binding of a ligand prior to protein folding can significantly accelerate the formation of functional protein [37]. Our *in vitro *experimental data show that apoNCS in its partially unfolded MG-like state resumes its native state after binding with NCS-Chr (Fig. 7). In our opinion, it may not be far-fetched to assume that in cellular environment, NCS-Chr binds apoNCS and effectively converts it into a functional protein for its own protection.

Recently, we have demonstrated apoNCS as a superior drug carrier, as its conformation is stable at wide pH range between 4–10 [7] and is highly resistant against organic solvents and chemical denaturants [35]. Here we further confirmed the superiority of apoNCS, as it exhibits high capability in binding and protecting the labile enediyne chromophore even under harsh acidic environment, where apoNCS conformation becomes intrinsically unstable and disrupted. How is NCS-Chr protected by the apoNCS unfolding intermediate is an interesting question. Based on the structural information obtained from NMR results (Fig. 6), residues T6, S10, S11, S14, and G16 at N-terminus, N60 and D58 at the loop region between the β-strand V (residues 53–57) and VI (residues 62–66), and S98, G104, G107, N113, and S111 at C-terminus are highly perturbed in the MG-like state of apoNCS. On the other hand, majority of the residues involved in the secondary structural interactions do not show appreciable chemical shift changes or broadening. Residues at the bottom of the chromophore binding cleft such as V34, G35, Q36, L45, G96, V95, and V108 show only small perturbation. In addition, the chemical shift of F52 and the disulfide bond C37-C47, both locate right below the nine-membered enediyne ring, are not significantly affected (Δδ of C47 is 0.094537 ppm, smaller than the 0.1 ppm threshold). It appears that many residues involved in the chromophore binding are not highly perturbed in the transition to the MG-like state. This probably accounts, at least in part, for the retaining ability of the MG-like state of apoNCS in binding and protecting the labile enediyne chromophore.

Besides being potent carrier of the natural ligand enediyne chromophore, apoNCS has also been demonstrated as a carrier of small synthetic molecules like EtBr [38], naphthoate ester derivatives [30,39,40] and flavone-based ligand [41]. Interestingly, apoNCS is useful in improving the stability of potent DNA alkylating agents, nitrogen mustards [40]. Furthermore, *in vitro *'evolution' studies revealed that apoNCS could be engineered into a common drug delivery vehicle [42]. Drug packaging for drug delivery systems has drawn extensive interests lately in the field of medicinal chemistry. Our study brings insight into the conserving mechanism of naturally occurring NCS and consequently merits apoNCS as a naturally built superior drug carrier for rational drug design strategies.

## Conclusion

We identified and characterized the stable MG-like state accumulated in the equilibrium early unfolding pathway of apoNCS in the presence of 1.2 M GdnHCl under acidic (pH 3) and aqueous conditions. The apoNCS intermediate retains about 80% and 40% of the secondary and tertiary structure, respectively. With the impaired binding cleft, the MG-like state of apoNCS still exhibits full capability of protecting the labile enediyne chromophore. The results demonstrate apoNCS as the natural built-in superior drug carrier. Further CD analyses showed that NCS-Chr not only binds to the MG-like state of apoNCS but also converts the partially unfolded protein to its functional native state of holoNCS for self protection.

## Abbreviations

NCS: neocarzinostatin; holoNCS: holoneocarzinostatin; apoNCS: aponeocarzinostatin; NCS-Chr: NCS chromophore; GdnHCl: guanidine hydrochloride; ANS: 1-anilino-8-napthalene sulfonate; MG: molten globule; CD: circular dichroism; C_m_: melting concentration; T_m_: melting temperature; SEC: size-exclusion chromatography; FPLC: fast protein liquid chromatography; HPLC: high pressure liquid chromatography; HSQC: heteronuclear single quantum coherence.

## Competing interests

The authors declare that they have no competing interests.

## Authors' contributions

AS carried out the research project and performed most of the study. TKSK conceived of the work, participated in the design of the study, interpreted the data and helped to draft the manuscript. CMH carried out HPLC and CD analyses for the drug protecting and binding experiments. CY supervised the project, helped in coordination, and provided essential instructions and directions. DHC participated in the design of the study, interpreted and critically reviewed the data, and made revision of the manuscript. All authors read and approved the final manuscript.
